# Arthroscopic Subtalar Arthrodesis after a Calcaneus Fracture Covered with a Forearm Flap

**DOI:** 10.1155/2011/930902

**Published:** 2011-01-13

**Authors:** Frederick Michels, Filip Stockmans, Stéphane Guillo, Jan Van Der Bauwhede, Dirk Oosterlinck

**Affiliations:** ^1^Department of Orthopaedics, AZ Groeninge, Burgemeester Vercruysselaan 5, 8500 Kortrijk, Belgium; ^2^Department of Orthopaedics, Bordeaux Mérignac Sports Clinic, 9, rue Jean Moulin, 33700 Mérignac, France

## Abstract

Surgical treatment of intraarticular calcaneal fractures is often associated with postoperative wound problems. Soft tissue necrosis, bone loss and uncontrollable infection are a challenge for the surgeon and amputation may in some cases be the ultimate solution. A free flap can be very helpful to cover a significant soft tissue defect and help in fighting the infection. However, the free flap complicates the surgical approach if subtalar arthrodesis and bone reconstruction are needed. This study demonstrates the value of an arthroscopic technique to resect the remaining articular cartilage in preparation for subtalar arthrodesis and bone grafting. This approach avoids compromising the soft tissues and minimizes damage to the free flap.

## 1. Introduction

Intraarticular calcaneal fractures are often associated with postoperative wound problems. Wound problems go hand in hand with infections, including deep infections that go down to the bone potentially leading to osteomyelitis. Uncontrollable infection or severely limited bone stock can preclude limb salvage, and amputation may be necessary [1, 2].

In case of a significant soft tissue defect, a microvascular flap can be used. The radial forearm free flap provides a quick, reliable, and easily harvested source of coverage for lateral heel wounds [3, 4]. However, this free flap and more precisely its feeding pedicle, complicates the classical anterolateral surgical approach if a subtalar arthrodesis is needed. This study analyses the value of an arthroscopically assisted approach to avoid compromise of the free flap.

## 2. Case Report

**Figure 1:**
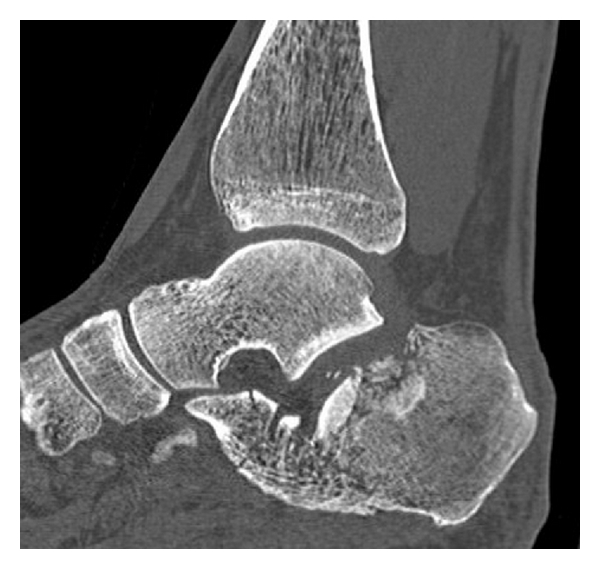
Initial sagittal computed tomography scan of the fractured calcaneus.

A 56-year-old male presented with a severe displaced intraarticular calcaneal fracture after a fall from a height (Figure 1). The medical history revealed significant tobacco abuse. 

After two weeks of elevation, an osteosynthesis was performed. One week postoperatively, serous drainage and erythema occurred and were treated with oral antibiotics and local wound care. Several weeks later serous drainage persisted originating from the apex of the L-shaped incision. Surgical debridements and vacuum-assisted closure (VAC) were used for several weeks to promote wound healing.

**Figure 2:**
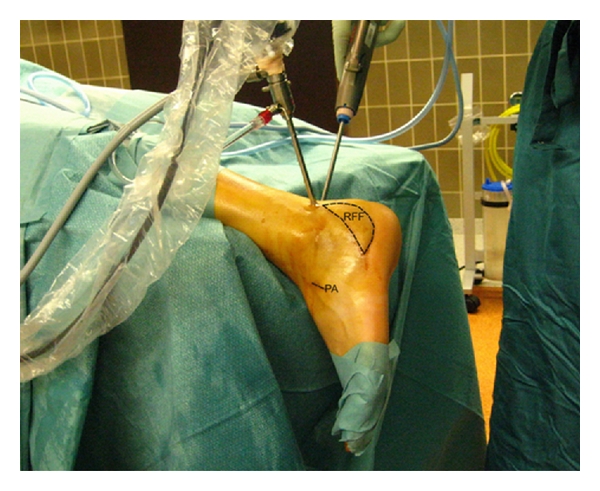
Peroperative view. Position of radial forearm flap covering the soft tissue defect, incision of anastomosis, and endoscopic portals: (RFF: radial forearm flap, PA: pedicle anastomosis).

Finally an osteomyelitis with significant avascular bone necrosis occurred. Culture results were positive for *S. aureus*. The implants were removed. An aggressive debridement was performed, removing all dysvascular bone and all infected, nonviable, or fibrotic tissues. The dead space was filled with an antibiotic-impregnated cement spacer [5]. The significant soft tissue defect was covered by a radial forearm free flap (Figure 2). The pedicle was anastomosed to the dorsalis pedis artery. No postoperative problems occurred.

After three months of oral antibiotic therapy, an arthrodesis was planned. The position of the free flap prevented a classical surgical approach. An endoscopic technique was used for subtalar arthrodesis. The patient was placed prone on the operating table with the foot and ankle extended slightly past the end of the table (Figure 2). No supports were used to allow a good view of the dorsal and lateral site of the ankle. This allowed an easier insertion of the bonegrafts. A 4.0-mm, 30-degree arthroscope was placed in a posterolateral portal. With a 3.5-mm shaver, introduced from a posteromedial portal, the articular surface of the posterior facet is debrided. A significant part of the calcaneus surface was missing since a former debridement. A small incision distally allowed removal of the cement spacer and insertion of iliac crest autografts. The subtalar arthrodesis was fixed with 2 percutaneously placed large, cannulated, 7.5-mm screws.

**Figure 3:**
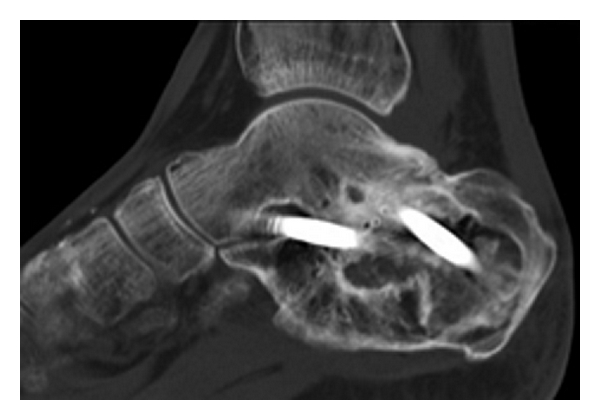
Sagittal computed tomography scan obtained 6 months postoperatively showing union of the posterior subtalar joint and incorportation of the bone grafts. Two large cannulated, 7.5-mm screws were used. The horizontally placed screw fixed the talar head, the other screw the talar body.

After 1 week cast, an ankle-foot orthosis was used during 12 weeks. Weight bearing was not allowed during 6 weeks. The patient healed without further wound problems. Fusion occurred after 12 weeks. A computed tomography scan performed 6 months postoperatively confirmed good incorporation of the bone grafts (Figure 3). After 1 year of followup the patient still had good function and no complaints.

## 3. Discussion

This case report illustrates the use of a radial forearm free flap and endoscopic techniques for reconstruction of soft tissues and bone after significant tissue defects. After osteomyelitis was controlled, a subtalar arthrodesis was performed.

The incidence of postoperative wound complications varies from 0 to 32.8% [1, 6]. Complications are higher in patients who are smokers, diabetics, have vascular disease, or have excessive swelling [1, 7]. The patient in this study had a history of significant tobacco abuse. As a result, he failed to stop smoking after his treatment started. Uncontrollable infection or severely limited bone stock can preclude limb salvage, and amputation may be necessary [1, 2, 6].

Osteomyelitis of the calcaneus often needs aggressive debridement and resection of all the nonviable structures. This treatment results very often in a significant tissue defect. The use of the radial forearm free flap to cover lateral heel wounds is reported with good results [3, 4, 8]. In some cases bone reconstruction and subtalar arthrodesis are necessary. With the pedicle steeled on the dorsalis pedis artery, a classical anterolateral approach for performing the arthrodesis is prevented. Consequently, an extensile posterolateral approach increases the risk of soft tissue damage and flap failure. To avoid new soft tissue problems, an endoscopically assisted technique was used.

The posterior portal approach for arthroscopic subtalar arthrodesis was first described by Van Dijk et al. in 2000 [9]. This technique has gained credibility in recent years because of several advantages. It is considered to be a safe technique that provides optimal visualization, a small incision, and limited dissection [10]. In this particular case, the endoscopic approach allows good resection of the articular cartilage and avoids compromising the soft tissues. Bone grafts were inserted using only a small approach. 

Performing reconstruction of soft tissue and bone in two episodes allows two difficult problems to be separated. The first intervention allows the eradication of infection and reconstruction of soft tissues. When the soft tissues have healed, bone reconstruction can be performed with an endoscopically assisted technique. An advantage of free-tissue transfer is that subsequent bone reconstruction is facilitated, as the increased vascularity in the recipient bed allows for the rapid incorporation of cancellous bone grafts [11].

## 4. Conclusion

This report describes how good results were obtained from the reconstruction of soft tissue and bone after an osteomyelitis of the calcaneus. A radial forearm free flap reconstruction was followed by an endoscopic subtalar arthrodesis. We recommend multidisciplinary management of significant tissue defects of the hindfoot. Soft tissue coverage is a challenge for the reconstructive microsurgeon. Experience in arthroscopic surgery helps the surgeon to perform further surgery and bone reconstruction without having to compromise the soft tissues once again.
